# Removal of As from Tambo River Using Sodium Alginate from *Lessonia trabeculata* (Aracanto)

**DOI:** 10.3390/plants14142173

**Published:** 2025-07-14

**Authors:** Diana M. Villanueva, Aldo G. Gonzales, Claudio A. Saez, Antonio M. Lazarte

**Affiliations:** 1 Laboratorio de Biotecnología Ambiental, Biomineria y Bioensayos Ecotoxicológicos LAB-BIOTBEC, Universidad Nacional de San Agustín de Arequipa, Santa Catalina 117, Arequipa 04000, Peru; agonzalescor@unsa.edu.pe (A.G.G.); alazarter@unsa.edu.pe (A.M.L.); 2 Departamento de Ciencias del Mar y Biología Aplicada, Universidad de Alicante, 03690 Alicante, Spain; claudio.saez@ua.es; 3 Hub Ambiental Upla-Facultad de Ciencias de Ciencias Naturales y Exactas, Universidad de Playa Ancha, Valparaíso 2360072, Chile

**Keywords:** sodium alginate, *Lessonia trabeculata*, arsenic

## Abstract

Arsenic (As) contamination in the Tambo River (Perú), linked to mining activities and volcanic eruptions, poses significant health and agricultural risks. This study evaluated sodium alginate extracted from the brown macroalgae *Lessonia trabeculata* (LT) as a biosorbent for As removal. Water samples from three river points revealed As concentrations up to 0.309 mg/L, exceeding regulatory limits (0.1 mg/L). Sodium alginate was obtained via a simplified alkaline method, yielding an average of 21.44% (*w*/*w* relative to dry algae biomass) and characterized by Fourier Transform Infrared Spectroscopy (FTIR), showing structural similarity to industrial alginate (A1). Biosorption assays under simulated environmental conditions (neutral pH, 20 °C) demonstrated that LT alginate (A2) reduced As by 99% at 48 h with a 1.0 g/L dose, outperforming A1. Langmuir (*q_max_* = 0.0012 mmol/g; *b* = 506.9 L/mg) and Freundlich (*n* = 1.94) isotherms confirmed favorable adsorption, while kinetics followed a Pseudo-Second-Order Model, suggesting physisorption. These results highlight LT alginate as a sustainable and scalable solution for remediating As-contaminated water, promoting the conservation of a vulnerable marine resource. This study underscores the potential of algal biopolymers in bioremediation strategies aligned with environmental and socioeconomic needs.

## 1. Introduction

Since 2017, numerous studies have been published on arsenic (As) removal processes from various liquid matrices [1]. This is due to the fact that As ranks among the ten most hazardous chemicals to human health [2]. The concentrations and relative proportions of As species can vary due to changes in input sources, biological activity, pH, and redox potential (Eh) [3], which determine the predominant As species [4]. As species include H_3_AsO_4_, H_2_AsO_4_^−^, HAsO_4_^2−^, and AsO_4_^3−^ for As (V) and H_3_AsO_3_, H_2_AsO_3_^−^, and HAsO_3_^2−^ for As (III) [5,6]. Given its toxicity, efficient removal methods such as biosorption with biopolymers have been extensively investigated.

This issue is particularly critical in agricultural contexts, where irrigation with As-contaminated water poses serious risks: As is absorbed by crops, accumulates in edible tissues, and enters the food chain, endangering human health [7,8]. Furthermore, As accumulation in agricultural soils alters edaphic microbiota, reduces essential nutrient availability (e.g., phosphorus, potassium), and decreases long-term crop productivity [9]. Such contamination poses a serious threat to the Tambo River, a vital agricultural water source for Arequipa, Peru, where elevated levels of As and other heavy metals originate from mining waste, excessive fertilizer application, pesticide runoff, and a range of environmental pressures [10,11,12].

In 2021, the National Water Authority (ANA) reported As concentrations as high as 0.377 mg/L at monitoring point 1318RTamb5 (October 2019), exceeding the national irrigation standard of 0.1 mg/L established by Supreme Decree N°004-2017-MINAM [13,14]. The problem intensified following the 2023 eruption of the Ubinas Volcano, whose ash emissions altered river chemistry by increasing suspended particles and elevating As, B, Hg, and Si concentrations [11]. These conditions have heightened the vulnerability of surrounding aquifers and raised alarm among local communities and authorities.

To address such contamination, this study explores the use of sodium alginate for biosorption, an eco-efficient, regenerative, and low-cost material. Biosorption involves mechanisms such as ion exchange, surface complexation, and physical adsorption, which vary depending on the biomass type, origin, and processing of the biomass used [15,16,17].

Alginate is a linear copolymer composed of homopolymeric blocks of β-D-mannuronic acid and α-L-guluronic acid, which are isomeric residues [18], extracted from brown algae through alkaline treatment [19]. This biopolymer exhibits structural variability, in fluenced by the algal species, harvest season, and extraction methodology. These factors affect monosaccharide sequences, glycosidic bond configurations, and block distributions (MM/GG/MG/GM), thereby influencing key properties like the carboxyl group (-COOH) orientation, chain packing, and molar mass [18]. The ionic cross-linking of its hydrogel structure (known as the “egg-box” model) enhances the biosorption capacity by capturing ions through charge interactions. While this ionic cross-linking (egg-box) mechanism is particularly effective for cations due to their affinity for alginate’s negative charges [20], As biosorption behavior differs under neutral pH conditions, where anionic species predominate. However, surface hydroxyl group protonation on alginate enables additional interactions that enhance As adsorption [21]. Despite these advantages, direct applications of sodium alginate remain limited.

To expand its use, alginate has been integrated into advanced technologies, such as packed-bed, isothermal, or steady-flow reactors, enabling potential industrial-scale application [22]. However, its mechanical performance is constrained by the proportion of G-blocks: concentrations exceeding 70% increase chain rigidity and length, while lower proportions favor elasticity. Still, alginate remains a renewable, reusable, and adaptable material with broad applicability across multiple industrial sectors and minimal functional degradation [18,23].

In this study, sodium alginate was extracted from *Lessonia trabeculata* (LT), a brown macroalgae endemic to the South American Pacific coast, particularly the rocky subtidal zones of Peru (2–20 m depth) [24,25,26]. LT has been employed as a biomonitor due to its capacity to sequester varying metal concentrations within its complex structure [27]. According to Leal et al., 2018 [19], sodium alginate extracted from *Lessonia* sp. exhibits a mannuronic/guluronic acid (M/G) ratio of 1.29 and 52% homopolymeric mannuronic (MM) blocks, characteristics that position it as an ideal biosorbent. The elevated mannuronic content confers a flexible and porous structure with accessible active sites (-COOH, -OH) for interaction with As [18]. The MM/GG block distribution further influences the negative charge density and ionic cross-linking capacity, a key mechanism in forming the “egg-box” network that traps ions [20]. While LT extraction supports artisanal fishing communities, recent overexploitation and informal harvesting have led to forest depletion, threatening marine ecosystem integrity [26]. Thus, valorizing LT is critical to promote conservation and raise local awareness.

This research evaluates the As biosorption performance of LT-derived sodium alginate, proposing it as a viable solution for the remediation of the Tambo River and similar contaminated aquatic systems, aiming to mitigate As pollution and enhance water quality.

## 2. Materials and Methods

### 2.1. Sampling Stations

**Figure 1:**
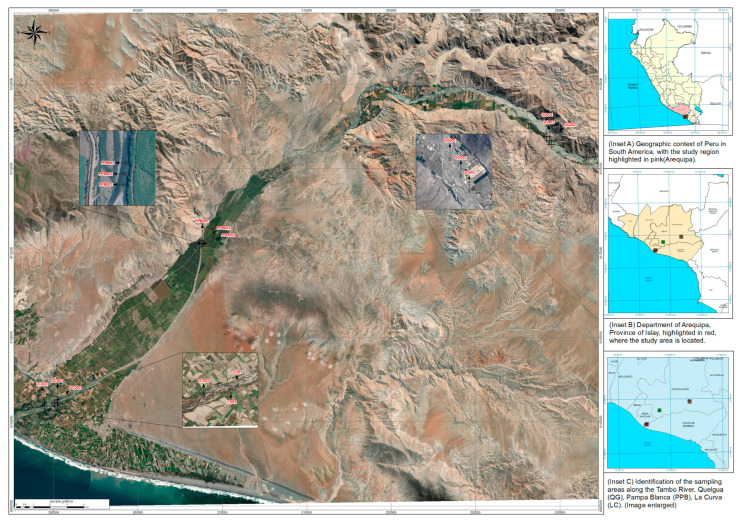
Geographic distribution of sampling points in the Tambo River, Islay, Arequipa.

Three water sampling points along the Tambo River were selected via satellite geographic assessment: upstream (before river entry into agricultural zones), midstream (within agricultural zones), and downstream (after agricultural zones). These corresponded to the populated centers of Quelgua Grande (QG00), Pampa Blanca (PPB00), and La Curva (LC00), respectively, as illustrated in Figure 1.

Site selection criteria included distance, vegetation cover, accessibility, river width/depth variations [28], seasonal changes (dry season: May–September; wet season: October–April), and anthropogenic impacts. Sampling protocols followed guidelines by Peru’s National Water Authority (ANA) [29,30]. A multiparameter probe (HANNA HI98194 (Hanna Instruments, Woonsocket, Rhode Island, United States)) was used to measure physicochemical water variables (Table 1). Total metal analysis, including As, was performed via ICP-MS following ISO 17294-2:2023 [30].

### 2.2. Sodium Alginate Extraction and Characterization from LT

**Figure 2:**
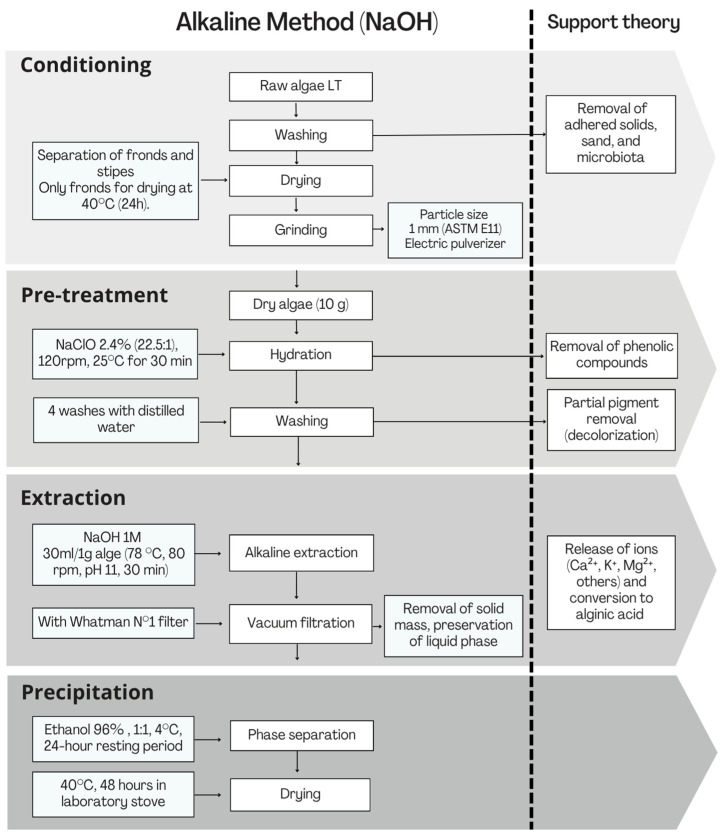
Flowchart of sodium alginate extraction from LT.

The brown macroalgae LT was used, with morphological validation performed using IMARPE (Peruvian Marine Research Institute) guidelines [31]. The extraction protocol is summarized in Figure 2.

Raw fronds were manually rinsed with distilled water under continuous agitation to remove sand, salts, epiphytes, and other adhered solids [32]. Selected algal fronds were dried in a forced convection oven [33], pulverized, and sieved through No. 18 (1.0 mm) and No. 20 (0.850 mm) mesh screens (particle size ≤ 1 mm), per ASTM E11 [32,34]. The sieved biomass was composed of monovalent (Na^+^, K^+^) and divalent (Ca^2+^, Mg^2+^, Sr^2+^, Ba^2+^) alginate salts [35], as treated with 2.4% NaClO solution [36]. This step oxidized organic compounds (e.g., pigments, non-alginate polysaccharides) as follows [36]:

(1) $$R-OH+NaClO\to Oxidation\;of\;organic\;compounds+{\left[Cl\right]}^{-}$$

Simultaneously, ion exchange displaced divalent cations, converting the alginate to its sodium form [37]:

(2) $$Ca,Mg,Sr,Ba\ldots -{Alg}^{+2}{\left[Na\right]}^{+}\to NaAlg+{\left[Ca\right]}^{+2}$$

NaClO also acted as a bleaching agent, removing natural pigments (e.g., brown algal phenols) and saponifying lipids [37]:

(3) RCOOR’+NaClO→RCOONa+R’OH

The alkaline method by Fawzy et al. (2017), Mohammed et al. (2018), and Nogueira et al. (2022) [38,39,40] was modified by omitting acidic pre-extraction. Direct alkaline extraction with 1M NaOH [41] enabled divalent cation displacement (Equation (2)) without intermediate double-displacement steps [42]. The solution was vacuum-filtered (Whatman No. 1 filter) to retain the liquid phase for ethanol precipitation [43]. The supernatant was recovered by filtration, and precipitated alginate was dried to a constant weight [35,44]. Yield (%) was calculated as

(4) $$Yield\;(\%)=\frac{Mass\;of\;extracted\;sodium\;alginate\;(g)}{Mass\;of\;dry\;algal\;biomass\;used\;(g)}*100$$

For spectral identification, the extracted alginate was analyzed using a PerkinElmer Frontier FT-IR/NIR spectrometer (ASTM E1252-98) [45], with a universal attenuated total reflectance (ATR) accessory. Wavenumbers ranged from 450 cm^−1^ to 4000 cm^−1^, excluding the 1700–2400 cm^−1^ region (accessory noise). Functional groups were identified using Spectragryph software (version 1.2.16.1), with spectral similarity assessed across three LT alginate replicates.

### 2.3. Alginate Interaction Assay

The objective of the assay was to evaluate the efficiency of LT sodium alginate (A2) compared to industrial sodium alginate (A1) in capturing As ions from a prepared standard solution (W1) and a Tambo River water sample (W2). The following experimental combinations were established in triplicate: T1:(A1 + W1), T2:(A2 + W1), T3:(A1 + W2), and T4:(A2 +W2).

The assay was conducted at a single concentration of [As] = 0.309 mg/L, corresponding to the latest recorded value from the zone of greatest impact (QG003). Based on this value, standard solutions of NaAsO_2_ (analytical grade) were prepared. All standard solutions were prepared using ultrapure water to avoid any interference from ions present in tap water.

Statistical significance was evaluated using two-way ANOVA with A1, A2, and the water matrix (W1 and W2) as independent factors, followed by Tukey’s HSD post hoc test (α = 0.05). Analyses were performed in R v4.4.1. Residual diagnostics confirmed model assumptions (normality: Shapiro–Wilk *p* = 0.934; homoscedasticity: Levene’s *p* = 0.386).

### 2.4. Langmuir and Freundlich Isotherms

The As biosorption isotherm was determined using the non-linear Langmuir and Freundlich Models, used to investigate the sorption equilibrium between metal ions and biomass layers [17,46,47,48].

The Langmuir Model suggests a monolayer absorption of the metal on a homogeneous surface, with uniform adsorption energies for all binding sites without any interaction between the adsorbed molecules [49,50] (Equation (5)):

(5) $$q_{e}=q_{max}\frac{C_{e}b}{\left(1+bC_{e}\right)}$$

where *q_e_* is the uptake of metal adsorbed at equilibrium in mol/g, *C_e_* is the concentration of the metal at equilibrium (mol/L), *b* is the constant related to the adsorption energy (expressed in L/mg), and *q_max_* is the maximum sorption amount of the biosorbent mol/g.

To evaluate adsorption, the equilibrium parameter is followed, calculating the dimensionless adsorption intensity (*R_L_*), where *C_i_* is the initial metal concentration in the solution in mol/g, expressed with Equation (6):

(6) $$R_{L}=\frac{1}{1+b\;C_{i}}$$

The average *R_L_* value found for different initial metal concentrations indicates a reversible process isotherm if equal to zero, unfavorable if equal to 1, and favorable if within the interval between 0 and 1 [49].

For the Freundlich isotherm, it starts from the premise of a heterogeneous energy distribution, implying different affinities for binding sites on the biomass surface, with interactions between adsorbed molecules and those with higher affinity [17,50], which are occupied first. The corresponding Equation (7) is

(7) $$q_{e}=K_{F}C_{e}^{\frac{1}{n_{F}}}$$

The Freundlich constants *K_F_* and *n_F_* are system-specific parameters determined by the adsorbate–adsorbent pair characteristics. The adsorption intensity, quantified by 1/*n_F_* (the slope of the linearized isotherm), serves as an indicator of process favorability according to Freundlich’s original criterion [51]: 1/*n_F_* > 1: weak adsorbate–adsorbent interactions (unfavorable adsorption); 1/*n_F_* < 1: strong interactions (favorable adsorption); and 1/*n_F_* = 1: linear partitioning behavior.

To evaluate the isotherms, the maximum adsorption capacity was determined using *q_e_*, expressed by Equation (8):

(8) $$q_{e}=\frac{C_{0}-C_{e}}{W}\;V$$

where *q_e_* is the amount of As adsorbed per alginate, *C_0_* is the initial concentration, *C_e_* is the equilibrium concentration expressed in mg/g, V is the solution volume (L), and W is the amount of adsorbent in grams [52].

Since A2 showed the highest As removal in the initial tests, we focused all isotherm and kinetic studies on A2 to understand its adsorption behavior and optimize its use.

### 2.5. Kinetics and Reaction Order

The reaction rate and mechanism were evaluated through time interval tests: 0, 10, 30, 60, 120, 240, 360, 480, 720, 1440, 2160, and 2880 min, reaching a 48 h period until equilibrium.

The Pseudo-Second-Order Model was applied to characterize the chemical interaction between adsorbates and functional groups on the adsorbent surface. Despite no significant optical changes over time, this method is effective [53,54]. The associated Equation (9) is

(9) $$\frac{t}{q_{t}}=\frac{1}{kq_{eq}^{2}}+\frac{1}{q_{eq}}\;t$$

where *q_eq_* is the amount of metal absorbed at equilibrium (mol/g), *k* is the sorption rate constant (g/mol·min), and *q_t_* is the amount of metal absorbed at time *t* (mol/g).

To predict the rate-limiting step, the Weber–Morris Intraparticle Model was used, analyzing solute adsorption relative to the square root of time [55,56,57]. This Equation (10) is expressed as

(10) $$q_{t}=k_{int}t^{\frac{1}{2}}+C$$

where q_t_ is the amount of solute adsorbed at time *t*, and *k_int_* is the intercept. Extrapolating from the initial linear portion of the plot, *C* is the intercept. Extrapolating from the initial linear portion of the plot, *C* relates to the boundary layer thickness [58].

A Weber–Morris plot of *t^(1/2)^* vs. *q_t_* determines whether intraparticle diffusion is the rate-limiting step. If the plot originates at zero, intraparticle diffusion is the sole rate-limiting step; otherwise, diffusion resistance arises from other factors [59].

The transformed experimental setup for interaction, isotherm, and kinetic evaluations is summarized in Table 2.

All tests were conducted in triplicate using a batch system. Temperature control was regulated via an orbital shaker incubator, and pH was maintained according to ambient conditions at sampling point QG00. Phase separation was achieved via polarity difference precipitation using 96% ethanol and solid–liquid decantation. Residual [As] was measured using a Bruker S2 Picofox Total Reflection X-ray Fluorescence (TXRF) spectrometer (Bruker Corporation, Billerica, Massachusetts, USA; Karlsruhe, Germany). For analysis, 1 mL of filtered solution was mixed with 10 μL of gallium standard solution, vortexed for 10 min, and 10 μL of the mixture was deposited onto quartz disks. The use of a gallium internal standard is required by the Bruker S2 Picofox TXRF system to normalize variations in sample deposition, matrix effects, and instrument drift, thereby ensuring accurate and reproducible quantification of As. Disks were sterilized with absolute acetone, 10% nitric acid, and SERVA silicone solution (SERVA Electrophoresis GmbH, Heidelberg, Germany) to create a smooth interface. Disks were heated at 55 °C on a hot plate, cooled, and analyzed for 1000 s.

## 3. Results and Discussion

### 3.1. Sampling Stations

The sampling zones exhibited pH variations between 6.50 and 8.24, and temperatures between 15.63 °C and 25.38 °C, values considered for controlling ex situ tests. ICP-MS analysis revealed that [As] was highest at QG00 and progressively decreased in the river estuary. These results indicate that dissolved [As] is elevated before entering agricultural areas, consistent with ANA reports documenting As levels exceeding 0.30 mg/L. Consequently, a threshold concentration of 0.309 mg/L was established for subsequent analyses.

The second sampling, conducted during the wet season, exhibited lower [As], suggesting that As is not diluted in the water column but rather sedimented as part of anaerobic degradation. The methanogenic activity of riverbanks involves a continuous accumulation of organic carbon sediments, which induce soil impermeability and CH_4_ production [60]. Resultant carbon isotopes promote the reductive dissolution of Fe (III) hydroxides and As-bearing oxides, enriching As in groundwater [60]. This phenomenon can also occur in surface waters, generating characteristic odors due to shoreline sediment disturbance, as observed during the second sampling campaign. These odors indicate the release of volatile compounds, such as hydrogen sulfide (H_2_S), resulting from anaerobic decomposition of organic matter in sediments. Furthermore, sediment removal during the wet season may expose deeper layers, releasing mineral-bound As and contributing to its mobilization into surface waters. This process accounts for the observed decline in aqueous As concentrations during the second sampling, as the metalloid was predominantly sequestered in sediments rather than diluted in the water column.

### 3.2. Extraction and Characterization of LT Sodium Alginate

Most studies employ calcium alginate in bead or reinforced matrix forms due to its aqueous stability [33,40]. In contrast, this work focused on A2, a biopolymer forming water-soluble gels that limit its direct application in aqueous systems [24,61]. The simplified alkaline extraction method with 1M NaOH omitted traditional protocol steps like acid pretreatment [35,36,37], utilizing 2.4% NaClO to remove pigments and unwanted organic compounds via oxidation and saponification (Equations (1) and (3)). The NaOH-based alkaline extraction yields are summarized in Table 3.

The proposed method for LT sodium alginate extraction achieved an average yield of 21.44% (ranging from 19.17% to 23.11%), reflecting the efficiency of recovery from dry biomass rather than suspended solutions. While this yield does not reach the 25% reported by Hernández-Carmona et al. (2012) [43,62] or the 17.30–30% range observed by Andriamanantoanina and Rinaudo (2010) [36], the method’s simplicity and reduced processing steps make it a practical and efficient alternative. The observed fluctuations in yield may stem from biomass homogeneity and experimental errors, yet the results demonstrate a reliable approach for sodium alginate extraction.

Compared to traditional methods (which involve acid/base pretreatments, bleaching, and precipitation), the simplified approach uses only conditioning, NaClO pretreatment, alkaline extraction, and C_2_H_6_O precipitation. This significantly reduces operational complexity, time, and resource requirements, proving reliable, reproducible, and scalable.

For characterization, A2’s FTIR spectra were compared to industrial alginate (A1) as a reference standard.

**Figure 3:**
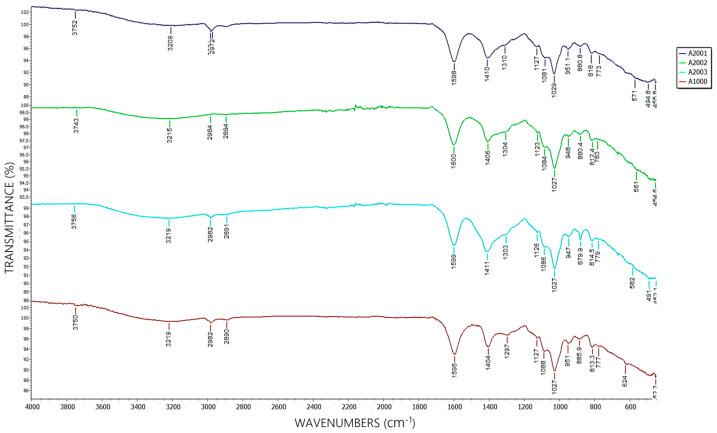
FTIR spectra of A2 compared to A1.

The characteristic functional-group bands detected by FTIR for A2 are compared to those of industrial alginate in Figure 3.

Figure 3 shows FTIR spectra of A2 and A1, identifying the following alginate-specific functional groups:

o Asymmetric COO^−^ stretching bands (1594–1600 cm^−1^), associated with uronic acid carboxyl groups (mannuronic and guluronic).
o Symmetric COO^−^ stretching bands (1410–1411 cm^−1^), confirming carboxylate presence.
o C-O-C glycosidic linkage peaks (1027–1029 cm^−1^), typical of alginate’s polysaccharide structure [61].

Additionally, bands between 1027 and 1083 cm^−1^ allowed the estimation of A2’s mannuronic/guluronic (M/G) ratio (~1.3). The higher intensity in this region suggests balanced M/G blocks, conferring optimal mechanical/gelation properties and an enhanced ion-exchange capacity [63].

Table 4 details the exact positions of functional groups and spectral similarity between A2 replicates (A2001, A2002, A2003) and A1. Similarity was calculated using the Pearson correlation coefficient across the full spectra (450–4000 cm^−1^), yielding values above 95% for all replicates. This indicates that A2 has a nearly identical chemical structure to industrial alginate, with minimal variations attributable to differences in biomass processing.

Spectral similarity (>95%) confirms that A2’s chemical structure is nearly identical to industrial alginate, validating its quality despite natural biomass variations. The alignment of functional group positions (R-COO^−^, OH^−^, C-O-C) between A2 replicates and A1 supports the method’s reproducibility and the biopolymer’s structural integrity.

### 3.3. Alginate Interaction Assessment

FTIR spectra confirmed that A2 exhibits a chemical structure consistent with A1. Based on this, the As biosorption capacity of both materials was evaluated under controlled conditions: neutral pH (7.0 ± 1.0), ambient temperature (20 ± 1 °C), and an initial As concentration of 0.309 mg/L (maximum recorded value at Tambo River’s QG00 point).

Assays were conducted in triplicate using a 0.31 mg/L alginate dose with constant agitation (120 RPM) for 24 h. Residual As concentrations were measured via X-ray fluorescence (XRF) spectrometry, and the results are shown in Table 5.

The high efficiency of A2 is attributed to its ability to interact with anionic As species (HAsO_4_^2−^/AsO_4_^3−^) through electrostatic interactions, where the COO^−^ groups of alginate attract negatively charged As ions (even at neutral pH, due to the partial protonation of hydroxyl (-OH) groups on the surface [21]), and via surface complexation, where As ions bind to active sites of alginate through coordination mechanisms, favored by its high solubility and dispersion in water. These results demonstrate that A2 requires no solid supports to achieve efficient As removal, highlighting its potential as a sustainable material for the remediation of contaminated waters.

Two-way ANOVA revealed highly significant main effects of both the alginate type (F(1.8) = 41.62, *p* < 0.001) and water matrix (F(1.8) = 28.81, *p* < 0.001), with no significant interaction between factors (F(1.8) = 2.65, *p* = 0.143). Post hoc analysis (Tukey’s HSD) showed that A2 (LT alginate) consistently outperformed A1 regardless of the water matrix, reducing residual As by 52.3% on average (*p* < 0.001); W2 enhanced As removal by 42.1% compared to W1 (*p* < 0.001); and the optimal combination (A2 + W2) achieved near-complete As removal (99.0%), significantly outperforming all other combinations (*p* < 0.05).

Despite structural similarities between A1 and A2, A2’s superior performance is attributed to its higher porosity and accessible binding sites, which remain effective in complex environmental matrices.

The absence of significant alginate–water matrix interaction (*p* = 0.143) demonstrates A2’s robustness across water types, a critical advantage for field applications where the water composition fluctuates seasonally [14]. This statistical confirmation of A2’s consistent performance underscores its practical utility for real-world remediation scenarios.

**Figure 4:**
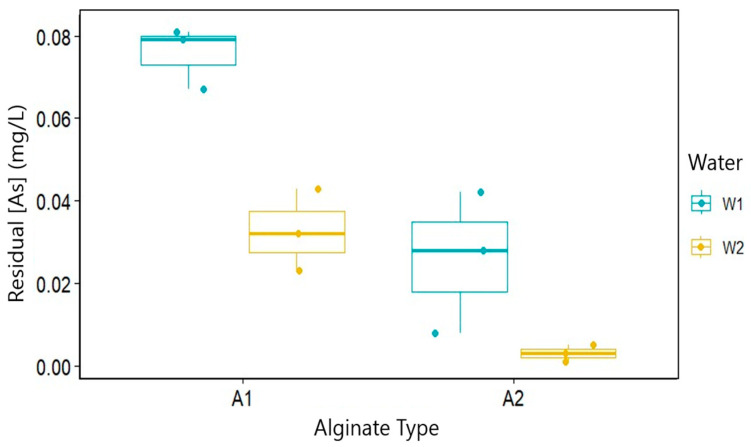
ANOVA effect of alginate type and water matrix on residual [As]. Blue boxes: W1; gold boxes: W2. A2 consistently outperformed A1, with optimal performance for W2. Different letters indicate significant differences (Tukey’s HSD, *p* < 0.05).

The results of the two-way ANOVA, illustrating the main effects of the alginate type and water matrix on residual [As], are depicted in Figure 4.

### 3.4. Langmuir and Freundlich Isotherms

After completing the contact time of A2 with W1 (at As concentrations of 0.2, 0.4, 0.6, 0.8, and 1.00 mg/L to evaluate capacities beyond theis study’s scope), the following results were obtained using the Langmuir equation. The Langmuir isotherm constants derived from the As adsorption experiments are summarized in Table 6.

From the regression of *C_e_/q_e_* vs. *C_e_*, the fitting parameters were obtained: *q_max_* = 0.00120739 and *b* = 506.9037. Here, *q_max_* represents the maximum adsorption capacity of sodium alginate, i.e., the maximum amount of As that can be adsorbed per unit mass of the adsorbent. A low value suggests that the material has limited capacity to retain As, which may depend on factors such as the porous structure, active surface area, or experimental conditions [64,65]. On the other hand, *b* reflects the interaction strength between As and the adsorbent. A high value indicates very strong affinity, meaning As is efficiently adsorbed even at low concentrations [64,66].

**Figure 5:**
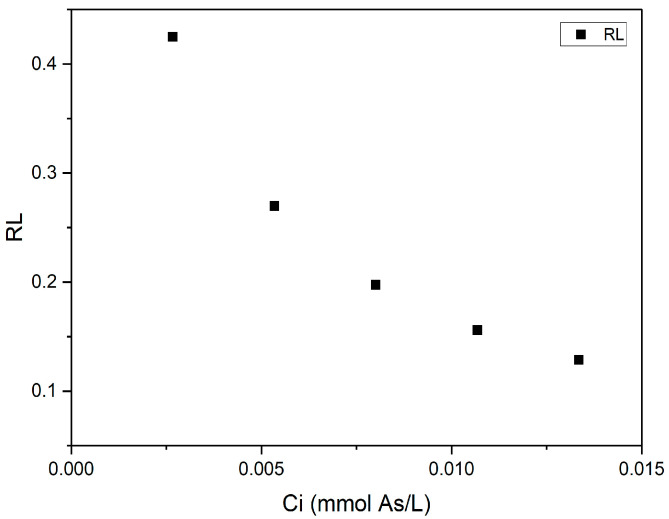
Langmuir Model fitting plot for As adsorption on A2.

To validate the Langmuir Model fit, the *R_L_* parameter was used, yielding a positive result (Figure 5).

The plot shows that As biosorption in sodium alginate is favorable at low initial concentrations (*C_0_*), evidenced by the decrease in the *R_L_* parameter as *C_0_* increases. The *R_L_* value remained within 0 < *R_L_* < 1, which, according to Lin 2002 [49], indicates beneficial and thermodynamically favorable behavior. This trend is explained by the Langmuir equation, where the high *b* = 506.9037 ensures that *R_L_* remains low even at elevated concentrations, reinforcing the material’s efficacy across a wide *C_0_* range.

The Freundlich Model was applied to characterize As adsorption on sodium alginate, yielding the following parameters: heterogeneity exponent (*n*) = 1.94476857 and capacity constant (*K_F_*) = 0.01378796 mmol/g. The transformed experimental data are presented in Table 7.

**Figure 6:**
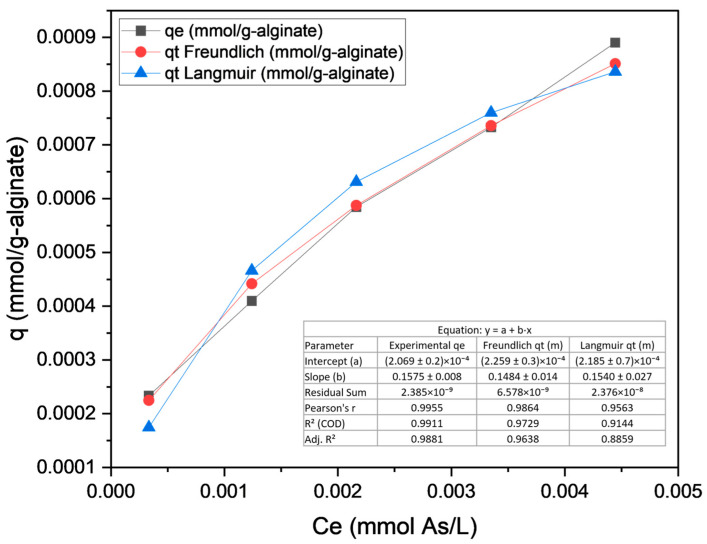
Comparison of Langmuir and Freundlich Models for As biosorption on sodium alginate.

The heterogeneity exponent *n* > 1 confirms a favorable As adsorption on sodium alginate, with a heterogeneous surface and variable-energy sites enabling contaminant uptake across a wide concentration range [67]. The proximity of *n* ≈ 2 suggests predominant physical interactions (physisorption), such as van der Waals forces, indicating potential reversibility. The moderate *K_f_* reflects the limited adsorption capacity, consistent with the Langmuir Model’s *q_max_* [68]. Linear regression (*log q_e_* vs. *log C_e_)* showed a high determination coefficient (*R^2^* > 0.98), validating the model fit and data consistency [69]. The combination of high *n* and low *K_f_* suggests that while alginate has As affinity, its efficiency could be optimized via structural modifications. The comparative fitting of Langmuir and Freundlich Isotherm Models to the experimental biosorption data is illustrated in Figure 6.

The Freundlich Model demonstrates a significantly better fit to the experimental data compared to the Langmuir Model, as evidenced by its higher correlation coefficient (*R^2^* > 0.98) and the parameter *n* = 1.94, which collectively suggest a heterogeneous adsorption surface and favorable physisorption conditions. Although Freundlich parameters (*K_f_*, *n*) are empirically robust, they lack a direct physical interpretation of adsorption sites [69]. In contrast, the Langmuir Model exhibits early saturation (*q_max_* = 0.0012 mmol/g), characteristic of homogeneous monolayer adsorption, but its inferior fit (*R^2^* < Freundlich) and deviation from the experimental data indicate limitations in describing this system’s complexity [64,65]. Thus, the Freundlich Model is more appropriate for characterizing these adsorption phenomena.

### 3.5. Kinetics and Reaction Order

Kinetic assays were conducted using the initial [As]_0_ from sample QG003, selected to represent a realistic contamination scenario prior to water entering agricultural zones. Table 8 shows residual [As]_e_ concentrations over time for three sodium alginate doses (0.1, 0.5, and 1.0 g/L), in triplicate for each.

**Figure 7:**
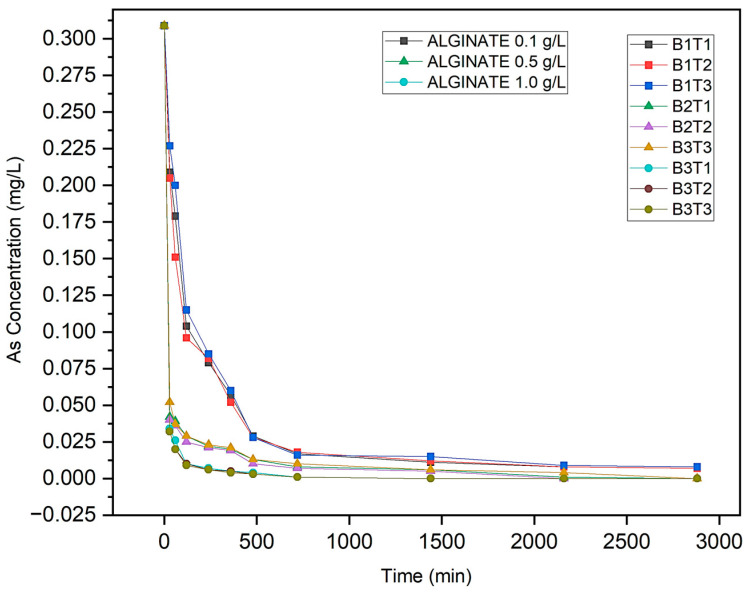
Concentration vs. reaction time for As biosorption.

[As]_e_ reduction showed a direct dependence on the alginate dose. At 1.0 g/L, >99% removal was achieved at 48 h (from 0.309 mg/L to <0.001 mg/L), with 90% As removed within the first 2 h. For 0.5 g/L, removal exceeded 95% at 24 h but required longer to reach near-zero values. At 0.1 g/L, efficiency was lower (~75% at 48 h), indicating that active site saturation limits the adsorption at lower doses. Experimental replicates (B1T1-B3T3) confirmed the reproducibility, with low dispersion (<5% standard deviation across doses), as shown in Table 8 and Figure 7.

Biosorption kinetics were analyzed using the Pseudo-Second-Order Model. Linearization of *t/q_t_* vs. *t* showed optimal fits for all doses (0.1, 0.5, 1.0 g/L), with *R^2^* > 0.99. Kinetic parameters (Table 9) revealed that the equilibrium capacity (*q_e_*) and rate constant (*k*) increased proportionally with the dose due to greater active site availability. As a dose of 1.0 g/L, the *q_e_* = 1.12 mmol/g and *k* = 0.045, while 0.1 g/L yielded *q_e_* = 0.41 mmol/g and *k* = 0.012. This increases reduced equilibrium time from 1500 min (0.1 g/L) to 120 min (1.0 g/L), demonstrating that higher alginate doses accelerate adsorption.

To complement the Pseudo-Second-Order Model (describing overall kinetics but not transport mechanisms), the Weber–Morris Model was applied. This identifies whether adsorption is limited by film diffusion (solute transfer from solution to adsorbent surface) or intraparticle diffusion (solute penetration into pores) [55].

The Weber–Morris results (Table 10) show a progressive q_t_ increase with *t_1/2_,* followed by adsorption capacity stabilization. For 1.0 g/L, *q_t_* rose from 0.369 mmol/g (*t_1/2_* = 5.48) to 0.412 mmol/g (*t_1/2_* = 53.67), indicating gradual active site saturation. This validates two stages: rapid surface adsorption (initial *t_1/2_*) and slower internal diffusion (higher *t_1/2_*), consistent with alginate’s porous nature.

**Figure 8:**
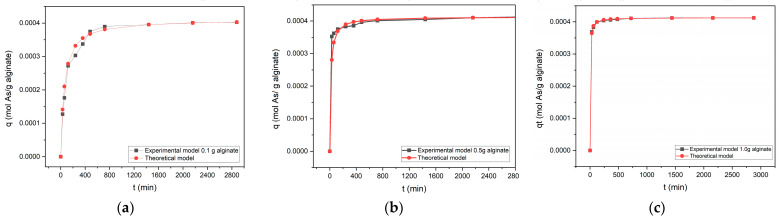
Pseudo-Second-Order Model applied to As biosorption kinetics using varying A2 doses: (**a**) 0.1 g, (**b**) 0.5 g, (**c**) 1.0 g. Each plot shows the relationship between the square root of time (*t^1/2^*) and adsorbed As (*q_t_*). The kinetic parameters derived from the Weber–Morris Intraparticle Diffusion Model are summarized in Table 11.

Figure 8 illustrates the *q_t_* vs. *t_1/2_*, curves, revealing two distinct regimes: a steep initial slope (*t_1/2_* < 10), linked to As diffusion through the liquid film toward the alginate surface, and a gentler slope (*t_1/2_* > 10), corresponding to intraparticle diffusion within the material’s pores. For the 1.0 g/L dose, the initial slope (0.35) indicates rapid mass transfer, while the reduced final slope (0.08) confirms internal diffusion as the kinetic bottleneck, consistent with McKay and Al-Duri’s findings [70]. This dual mechanism explains the delayed equilibrium in systems with a high active site density, despite rapid initial adsorption.

Alginate’s porous structure, capable of forming ion-trapping gels [71], explains its efficacy in As removal. However, performance varies with physicochemical properties such as molar mass and synthesis methodology. While the Pseudo-Second-Order Model adequately describes kinetics (*R^2^* > 0.99), the process complexity necessitates complementary models like Weber–Morris to identify rate-limiting steps.

## 4. Conclusions

This study demonstrates that sodium alginate extracted from LT is an effective biosorbent for As removal from contaminated water, achieving an average yield of 21.44% by alkaline extraction and exhibiting structural characteristics comparable to industrial alginate. While industrial alginate (A1) provided a valuable benchmark for structural comparison, LT alginate (A2) demonstrated an unequivocally superior As removal capacity (99.0% vs. ≤89.3%). At a dose of 1.0 g/L, A2 achieved a 99% As reduction within 48 h. Subsequent isotherm and kinetic analyses confirmed A2’s favorable adsorption characteristics, with the Langmuir and Freundlich Models showing optimal parameters (*R_L_* between 0 and 1; *n* = 1.94), high affinity (*b* = 506.9 L/mg), and a maximum capacity (*q_max_* = 0.0012 mmol/g), albeit constrained by the material’s structure. Furthermore, Kinetics fitted to the Pseudo-Second-Order Model confirm that physisorption was the predominant adsorption mechanism, supported by interactions between alginate’s functional groups and As ions. These comprehensive results establish A2 as the sole candidate for scaled implementation. These results highlight that As biosorption using LT not only provides a viable technical solution for contamination mitigation but also promotes the sustainable valorization of LT, underscoring the potential of algal-derived biopolymers in bioremediation.

## Figures and Tables

**Table 1:** Physicochemical parameters and As levels at sampling sites.

Zone	Date	Hour (h)	Length (m)	Depth (m)	GPS Coordinates		Altitude m.s.n.m.	pH	ppmDO	uS/ cm	ppm Tds	Temp °C	Press. kPa	[As] mg/L
QG001	2 July 2022	10.34	26.75	0.84	17°00′59″ S	71°32′36″ W	334	7.74	34.24	1313	660	15.63	97.63	0.313
QG002	11 December 2022	9.10	17.45	0.67	17°01′07″ S	71°32′31″ W	335	6.5	26.15	595	476	21.83	101.08	0.127
QG003	22 August 2023	10.30	38.41	0.78	17°01′14″ S	71°32′27″ W	342	7.45	29.87	2465	1233	20.92	100.39	0.309
PPB001	2 July 2022	12.02	15.46	1.04	17°04′15″ S	71°44′08″ O	94	7.08	31.09	1563	782	18.58	100.8	0.229
PPB002	11 December 2022	11.32	15.46	0.58	17°04′16″ S	71°44 ′09″ O	94	6.51	13.87	405	891	25.38	101.28	0.075
PPB003	22 August 2023	13.10	17.20	0.76	17°04′17″ S	71°44 ′09″ O	93	7.2	24.66	227	214	21.64	101.49	0.266
LC001	2 July 2022	14.30	11.87	0.98	17°09′18″ S	71°49′03″ O	12	6.77	33.75	1641	821	21.72	101.63	0.168
LC002	11 December 2022	13.34	11.13	0.44	17°09′20″ S	71°49′08″ O	12	6.53	10.03	1866	937	22.78	101.08	0.106
LC003	22 August 2023	15.24	10.87	0.53	17°09′24″ S	71°49′29″ O	13	6.89	28.92	3600	1838	23.91	97.63	0.129

**Table 2:** Summary of experimental details for interaction, isotherm, and kinetics.

Parameters	Alginate Interaction	Isotherm	Kinetics
Mass (g)	0.5	0.5	1–0.5–0.1
Volume (mL)	100	100	100
Time (min)	1440	1440	0–10–20–30 60–120–240 360–450–720 1440–2160–2880
RPM	120	120	120
Temperature °C	20 (±1)	20 (±1)	20 (±1)
Neutral pH	7 (±1)	7 (±1)	7 (±1)
[As] W1 mg/L	0.31	0.2–0.4–0.6–0.8–1.00	---
[As] W2 mg/L	0.309	---	0.309

"---": Analysis not performed for this specific assay.

**Table 3:** Yield results for A2 extraction.

Performance Results				
Replicates	Code	Mass		Yield (%)
		LT Algae (g)	Alginate Obtained (g)	
1	A2001	10.000	2.204	22.040%
2	A2002	10.000	2.311	23.114%
3	A2003	10.000	1.917	19.166%

Yield values from three replicates of the extraction procedure using 10 g of dry LT biomass. Yield (%) was calculated as the ratio of extracted alginate mass to initial dry algae mass. Average yield: 21.44%, with minor variation between replicates.

**Table 4:** Functional group positions and spectral similarity between A2 and A1.

A2 Replicates	Functional Group Similarity			Spectral Similarity to A1 (%)
	R-COO^−^	OH^−^	C-O-C	
A2001	1594	1410	1029	99.340%
A2002	1598	1406	1027	97.300%
A2003	1600	1411	1027	96.690%
A1000	1599	1404	1027	100.000%

Industrial standard (A1).

**Table 5:** Residual [As] (mg/L) after biosorption with A1 and A2.

Sample	Description	Replicate 1	Replicate 2	Replicate 3	Average ± SD	Reduction (%)	Statistical Grouping
T1	A1 + W1	0.081	0.067	0.079	0.076 ± 0.007	75.4	c
T2	A2 + W1	0.042	0.028	0.008	0.026 ± 0.015	91.6	b
T3	A1 + W2	0.032	0.043	0.023	0.033 ± 0.010	89.3	b
T4	A2 + W2	0.001	0.005	0.003	0.003 ± 0.002	99.0	a

T1 and T2: Evaluate A1 and A2 efficiency in W1. A2 showed 91.6% reduction, exceeding the permissible irrigation limit (0.1 mg/L [8]). T3 and T4: Assess performance in W2. A2 achieved 99% removal despite competing ions and organic matter interference. Different lowercase letters indicate significant differences (Tukey HSD test, *p* < 0.05). Groups: a (highest efficiency) > b > c (lowest).

**Table 6:** As adsorption: Langmuir parameters with alginate.

[As]_0_ (mg/L).	[As]_e_ (mg/L)	[As]_0_ (mmol/L)	[As]_e_ (mmol/L)	*q_t_* (mmol/g-Alginate)	*q_e_* (mmol/g-Alginate)	*C_e_/q_e_*
0.2	0.025	0.00266951	0.00033369	0.00017468	0.00023358	1.42857143
0.4	0.093	0.00533903	0.00124132	0.00046631	0.00040977	3.02931596
0.6	0.162	0.00800854	0.00216231	0.00063137	0.00058462	3.69863014
0.8	0.251	0.01067806	0.00335024	0.00075992	0.00073278	4.57194900
1	0.333	0.01334757	0.00444474	0.00083624	0.00089028	4.99250375

Results indicate that equilibrium adsorption capacity (*q_e_*) increases with [As]_0_, from 0.000234 to 0.000890 mmol/g of alginate, while the *C_e_/q_e_* ratio rises from 1.43 to 4.99, demonstrating a linear correlation consistent with the Langmuir Model. This validates its applicability (monolayer adsorption) and confirms alginate’s effectiveness for As removal.

**Table 7:** Initial and final As concentrations converted to Freundlich equation.

[As]_0_ (mg/L)	[As]_e_ (mg/L)	*C_e_ calc* (mmol/L)	[As]_0_ (mmol/L)	[As]_f_ (mmol/L)	*q_t_* (mmol/g- Alginate)	*q_e_* (mmol/g- Alginate)	*log q_e_*	*log C_e_* Final
0.2	0.025	0.1228	0.00267	0.0003337	0.0002248	0.00023358	−3.63156	−3.4766578
0.4	0.093	0.164	0.005339	0.0012413	0.00044175	0.00040977	−3.38746	−2.9061148
0.6	0.162	0.1969	0.008009	0.0021623	0.00058764	0.00058462	−3.23312	−2.6650828
0.8	0.251	0.2211	0.010678	0.0033502	0.00073603	0.00073278	−3.13503	−2.4749241
1	0.333	0.2444	0.013348	0.0044447	0.00085118	0.00089028	−3.05047	−2.3521535

Experimental [As]_0_ and [As]_e_ in solution, expressed in mg/L and mmol/L. Adsorption data (*q_t_*, *q_e_*) in mmol/g alginate and logarithmic values (*log q_e_*, *log C_e_*) are included for Freundlich Model analysis. The third column (*C_e_ calc*) gives the equilibrium concentration predicted by the Freundlich isotherm, using *K_F_* = 0.01378796 and *n* = 1.9448. All values are presented in both mg/L (or mmol/L) and mmol/g, and the corresponding *log q_e_* and *log C_e_* transformations are included to facilitate linear regression analysis and assessment of model consistency.

**Table 8:** As biosorption kinetics for A2 and W2.

Time	Alginate 0.1 g			Alginate 0.5 g			Alginate 1.0 g		
(min)	[As] mg/L			[As] mg/L			[As] mg/L		
	Replica 1 B1T1	Replica 2 B1T2	Replica 3 B1T3	Replica 1 B2T1	Replica 2 B2T2	Replica 3 B2T3	Replica 1 B3T1	Replica 2 B3T2	Replica 3 B3T3
0	0.309	0.309	0.309	0.309	0.309	0.309	0.309	0.309	0.309
30	0.209	0.205	0.227	0.042	0.04	0.052	0.034	0.032	0.032
60	0.179	0.151	0.200	0.039	0.036	0.037	0.026	0.020	0.020
120	0.104	0.096	0.115	0.029	0.025	0.029	0.010	0.010	0.009
240	0.079	0.082	0.085	0.022	0.021	0.023	0.007	0.006	0.006
360	0.057	0.052	0.060	0.020	0.019	0.021	0.005	0.005	0.004
480	0.029	0.028	0.028	0.013	0.010	0.013	0.004	0.003	0.003
720	0.017	0.018	0.016	0.008	0.007	0.010	0.001	0.001	0.001
1440	0.011	0.012	0.015	0.006	0.005	0.006	0.000	0.000	0.000
2160	0.008	0.008	0.009	0.001	0.000	0.004	0.000	0.000	0.000
2880	0.007	0.007	0.008	0.000	0.000	0.000	0.000	0.000	0.000

Table 8 presents As biosorption kinetics using different alginate doses (0.1 g, 0.5 g, 1.0 g). [As] (mg/L) is shown as a function of time (min), with three replicates per condition.

**Table 9:** Pseudo-Second-Order Model parameters.

Dose A2 (g/L)	q_e_ (mmol/g)	k (g/mmol·min)	R2
0.1	0.41 ± 0.02	0.012 ± 0.001	0.994
0.5	0.89 ± 0.03	0.028 ± 0.002	0.998
1.0	1.12 ± 0.05	0.045 ± 0.003	0.997

Table 9 summarizes parameters from fitting experimental data to the Pseudo-Second-Order Model. Includes dose (g/L), equilibrium capacity (q_e_, mmol/g), rate constant (k), and R^2^. Values include standard deviations.

**Table 10:** Weber–Morris Model results.

t_1/2_	q_t_ Exp	t_1/2_	q_t_ Exp	t_1/2_	q_t_ Exp
0	0	0	0	0	0
5.47722558	0.12710002	5.477225575	0.35267396	5.47722558	0.36869105
7.74596669	0.17648603	7.745966692	0.36246218	7.74596669	0.38292846
10.9544512	0.27214362	10.95445115	0.37536483	10.9544512	0.39939046
15.4919334	0.30284303	15.49193338	0.38292846	15.4919334	0.40383965
18.973666	0.3371018	18.97366596	0.38559797	18.973666	0.40606425
21.9089023	0.374475	21.9089023	0.39627603	21.9089023	0.40784392
26.8328157	0.38960224	26.83281573	0.40117014	26.8328157	0.41095836
37.9473319	0.39538619	37.94733192	0.40472949	37.9473319	0.41229311
46.4758002	0.40117014	46.47580015	0.41006852	46.4758002	0.41229311
53.6656315	0.40250489	53.66563146	0.41229311	53.6656315	0.41229311

Table 10 shows Weber–Morris Model results for intraparticle diffusion analysis. Includes *t^1/2^* and q_t_ for three experimental datasets.

**Table 11:** Weber–Morris Model parameters.

Dose A2 (g/L)	Initial Slope (mmol/g·min^1^/^2^)	Final Slope (mmol/g·min^1^/^2^)
0.1	0.15 ± 0.01	0.02 ± 0.001
0.5	0.28 ± 0.02	0.05 ± 0.003
1.0	0.35 ± 0.03	0.08 ± 0.004

Table 11 summarizes parameters from the Weber–Morris Model for intraparticle diffusion analysis in As biosorption. Initial and final slopes (mmol/g·min^1^/^2^) are shown for A2 doses (0.1 g/L, 0.5 g/L, 1.0 g/L), with standard deviations.

## Data Availability

The original contributions presented in this study are included in the article. Further inquiries can be directed to the corresponding author.

## Abbreviations

The following abbreviations are used in this manuscript:

As	Arsenic
LT	*Lessonia trabeculata*
A1	Industrial sodium alginate
A2	Sodium alginate extracted from *Lessonia trabeculata*
W1	Prepared standard solution
W2	Tambo River water
FTIR	Fourier Transform Infrared Spectroscopy
ICP-MS	Inductively Coupled Plasma Mass Spectrometry
q_max_	Maximum adsorption capacity (Langmuir Model)
C_e_ calc	Calculated equilibrium concentration based on the Freundlich model equation
R_L_	Langmuir equilibrium parameter
K_F_	Freundlich constant
n_F_	Heterogeneity exponent (Freundlich Model)
RPM	Revolutions per minute
DO	Dissolved oxygen
TDS	Total dissolved solids
PSI	Langelier Saturation Index (calculated from pH, TDS, and temperature)
ANA	National Water Authority (Peru)
CH_4_	Methane
Fe(III)	Iron in the +3-oxidation state
